# Design and test–retest reliability of a new test to assess motor competence in healthy older adults

**DOI:** 10.1007/s11357-025-01812-5

**Published:** 2025-07-26

**Authors:** José Carlos Cabrera Linares, Pedro Ángel Latorre Román, Manuel Lucena Zurita, Juan Antonio Párraga Montilla

**Affiliations:** 1 Department of Musical, Plastic and Corporal Expression, University of Jaén, Paraje las Lagunillas, S/N, 23071 Jaén, Spain; 2 Escuelas Profesionales de la Sagrada Familia, 23400 Úbeda, Jaén Spain

**Keywords:** Older adults, Motor competence, Gross motor skills, Fine motor skills

## Abstract

The objective of this study was to design and analyze the test–retest reliability of a test to assess holistic motor competence in older adults (HMCT-OA), as well as the capacity of this test to discriminate between sex and age. A total of 239 older adults (age = 72.44 ± 5.93 years old) joined in this study. The test was divided into motor cognitive section, locomotor section, and manipulative section. Each section and the total time to complete the test was registered. Intraclass correlation coefficient analysis showed 0.997 (95% CI = 0.996–0.998; *p* < 0.001) for the total test. Also, a strong correlation between the total test and each section was found. No significant differences between sexes were found; however, the participants over 80 years old showed worse performance. The HMCT-OA showed excellent test–retest reliability parameters for older adults. The test is safe, easy to set up, and was also able to discriminate per age in healthy older adults.

## Introduction

Nowadays, Spain is one of the European countries with the highest levels of life expectancy at birth (82.4 years). This entails a demographic revolution, which poses new challenges in the field of health, sustainability, and personal autonomy [1]. Although populations around the world are quickly aging, this increase of longevity is not being accompanied by a prolonged period of good health, therefore, a redefinition of healthy aging is necessary that emphasizes the concept of functional capacity [2].

Aging has been associated with frailty and functional limitation due to three factors: an irreversible biological process, deconditioning due to a sedentary lifestyle, and the effects of comorbidity [3]. Particularly, in older adults, motor performance deficits could be due to dysfunction of the central and peripheral nervous systems as well as the neuromuscular system [4].

There are several consequences of the alteration of motor unit morphology and properties on motor function of older adults, affecting different aspects of motor performance such as strength, power, contraction velocity, fatigability, and force steadiness [5]. Also, reaction time, low ability to control and execute movements, and difficulties in learning new motor skills [6], which lead to a large intra- and interindividual variability in many aspects of motor performance that increase with advanced aging [5]. With regard to this, older adults tend to walk more slowly, have reduced muscle strength, and show declines in memory and reasoning abilities. They also exhibit slower responses in speeded cognitive tasks compared to younger adults and to their own performance at earlier ages [7]. Therefore, gross motor skills which are used when moving from one place to another, climbing stairs, or avoiding any obstacles can decrease as the age increases [7, 8].

Quantitative biomarkers of aging are valuable tools to measure physiological age, evaluate the extent of “healthy aging”, and potentially predict health and life span for an individual. Being physical function and anthropometry the most practical measurements among phenotypic biomarkers of aging [9]. Especially, measuring physical capacity is a feasible method to identify accelerated aging and biological age [10]. Functional assessments for physical performance such as handgrip strength, chair stand, gait speed, complex gait, timed up and go, standing balance times, and 6-min walk tests are frequently used for monitoring the biological aging process since these tests are predictors of all-cause mortality and survival in older community-dwelling populations [11, 12]. In particular, motor competence (MC) could be a relevant phenotypic marker of health throughout life [13].

In this regard, physical function, expressed through MC, understood as the level of development of basic motor skills, is essential for performing most activities of daily life, work, personal autonomy, and leisure enjoyment, all of which align with physical functioning. In this sense, the systematic practice of physical activity leads to improve physical fitness and MC through a complex system of reciprocal interactions, enhancing individual’s overall health. Stodden et al. [14] established a conceptual model describing the relationships between physical activity, MC, physical fitness, obesity, and health. The model emphasizes that MC development is a primary underlying mechanism promoting participation in physical activity, with clear implications for health promotion. In this regard, current evidence indicates that MC is associated with health, correlates with physical activity levels [15], physical fitness [16], and weight status [17], as well as psychosocial health, cognitive functioning, and academic performance [18].

Several studies have shown that moderate physical activity reduces mortality and morbidity, and improves quality of life [19–21]. In addition, older adults with higher physical fitness and higher physical activity levels show more efficient brain activity and higher executive function [22]. Especially, older adults need to keep a proper MC level since it allows them to have greater independence (e.g., to move from one place to another on their own) [23]. Also, to avoid the risk of falls, which is one of the major causes of mortality at these ages [24]. In this regard, the relationship between MC and physical activity has been widely studied in the young population [25, 26]. Nevertheless, the number of studies that have adequately analyzed and evaluated MC in older adults remains limited [27, 28].

According to Hulteen et al. [29], the MC assessment has been used in multiple studies in child, adolescent, and young adult populations [18, 30, 31]; however, evidence across the entire adult age spectrum and in older adults is not yet available. In general, there has not been a focus on MC assessment in older adulthood (60 + years). In this sense, one of the limitations in studying and improving MC is the necessity for valid and reliable tests that allow researchers and professionals to quantify MC levels, identify skill deficiencies, and determine the effectiveness of interventions in developing motor skills [29].

Nowadays, several tests are used to assess MC in older adults [32]; nevertheless, these tests were not specifically designed for this population since they were designed to evaluate MC in a wide range of populations [8]; are based on observational scale [33]; have partially evaluated, through specific tests, certain components of MC such as balance, throwing, and walking [34, 35]; and have been designed to evaluate performance in daily living activities, primarily used to identify individuals at risk of disability or loss of functional independence. Additionally, many of these tests cannot be universalized as they involve motor skills that are not culturally transferable. The lack of ecological validity or the bias of qualified observation in qualitative assessments are additional limitations of the tests currently used to evaluate MC [36].

MC is evaluated through product- or process-oriented assessments. Product-oriented assessments emphasize on quantitative results of the task without assessing how a movement is executed which is analyzed from the perspective of the process, based on the qualitative aspects of the movement patterns [36]. Product measures are fundamentally more objective and may be the most advantageous alternative for examining changes in MC over time and reducing bias regarding the establishment of intra- and inter-rater reliability scores. However, measuring performance using product scores may be limited by access to technology [36]. Moreover, in aging research, it is necessary to develop tests that include cognitive challenges and mirror everyday demands; thus, environmental constraints and motor-cognitive demands should be inherently integrated into assessments of MC [36].

Currently, there is no international agreement on a reference test (gold standard) for evaluating MC in healthy older adult populations. Therefore, a quantitative and holistic evaluation of both locomotor and manipulative skills in a dual context would provide more precise information on the evolution of MC in older adult populations. Also, this test should emphasize on perceptuomotor integration in dynamic performance conditions (decision-making, force regulation, speed, and accuracy…) [36]. In addition, there are other factors such as the time required to conduct the test, the specific equipment, the organization of the results, the interpretation of the results [32], also monetary (equipment, research needed…), and non-monetary (time-consuming) cost [37] have to be kept in mind when an ecological test is designed.

Therefore, the objective of this study was to design and analyze the test–retest reliability of a test to assess MC in older adults, as well as the capacity of this test to discriminate between sex and age.

## Methods

### Participants

This cross-sectional study was composed of a total of 239 older adults; (174 women; mean age = 72.44 ± 5.93 years old; BMI: 21.70 ± 3.69 kg/m^2^). The participants were recruited at various leisure centers for older adults in the south of Spain (Andalusia), and they were classified into different age groups (i.e., group 1: 60–69 years, Group 2: 70–79 years, Group 3: + 80 years) according with previous studies [39, 40]. The inclusion criteria were as follows: (a) Older than 60 years old; (b) independent ambulation; (c) free of any disease that requires drugs daily that could affect gait performance;(d) do not have a diagnosis of any disease associated with a risk of falls; (e) the participants were not institutionalized in any care homes. All the participants signed an informed consent before to join in this study. Moreover, this study was conducted attending to the norms of the Declaration of Helsinki (2013). Also, the study was approved by the Ethics Committee of the University of (omitted to avoid any author’s identification).

### Material and testing

#### Holistic motor competence test

**Fig. 1:**
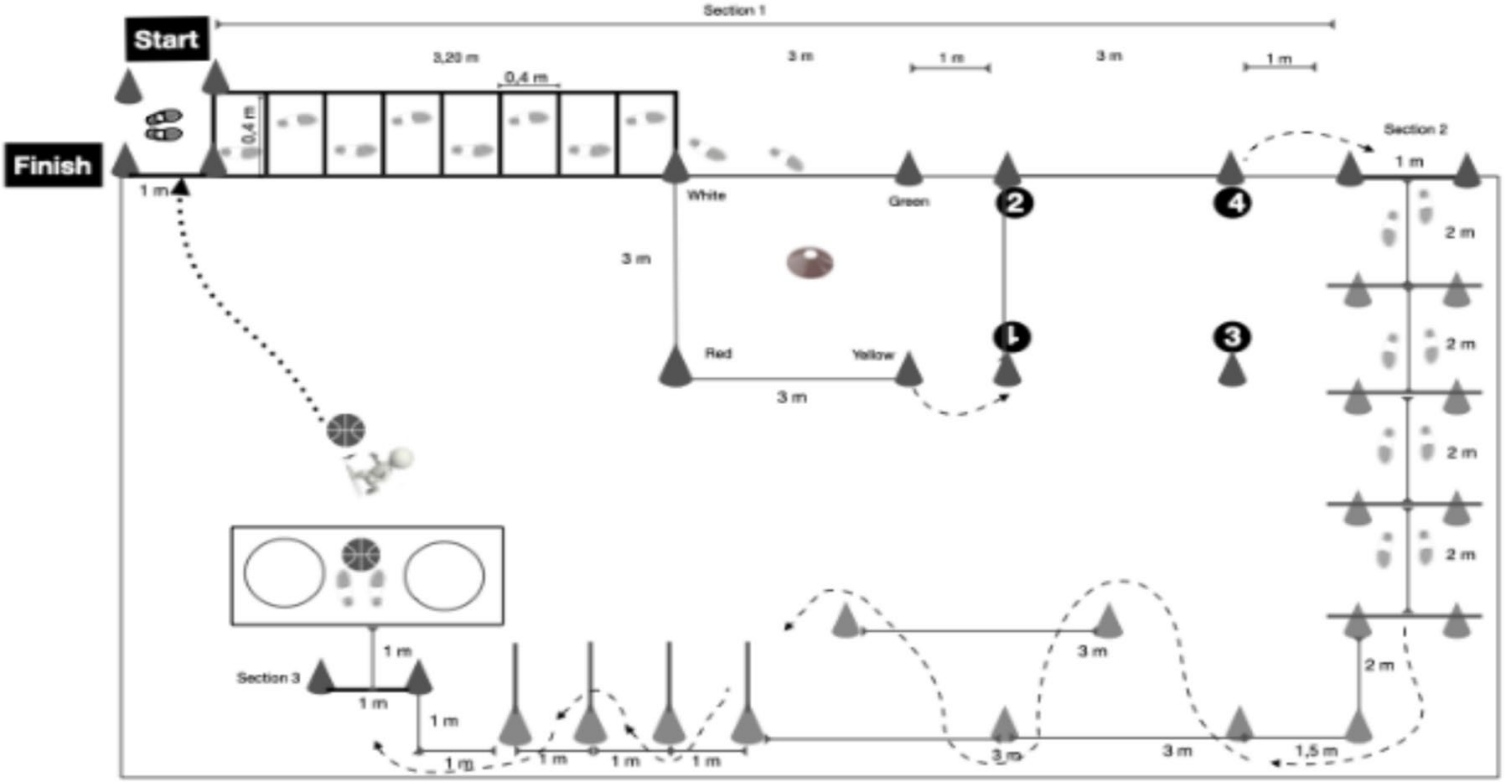
Holistic motor competence test (HMTC-OA)

The Holistic Motor Competence Test in Older Adults (HMCT-OA) (Fig. 1) test had a total distance of 13 m × 15 m, and it was divided into three different sections. The total time to complete the test was registered (i.e., Section 1 + Section 2 + Section 3). Also, the time to complete each section separately was registered (Sections 1, 2, and 3). Each section was designed to assess different aspects of MC. The first section evaluated motor-cognitive abilities, decision-making capacity, and fine motor skills. The second section focused on the assessment of participants’ locomotor competence. The third section examined manipulative skills, including gross motor manipulation using both the hands and the legs.

#### Motor-cognitive section

Right from the start point, the participant had to walk/run through a speed ladder composed of 8 squares of 40 × 40 cm pointed out on the ground floor being compulsory to step inside each of the squares (alternatively) with at least one foot (i.e., left–right-left…). Subsequently, the participant went inside a square (3 × 3 m) delimited by cones (white, yellow, red, and green). In the center of the square, placed on the ground, four training cones (a cone with a hole in the middle so it can be inserted into another cone) with the same color as the external cones were situated. The participant had to take each training cone individually (fine motor skills) and put it inside the cone with the same color (i.e., white-white, yellow-yellow, and so on).

Once, the last cone was placed, the participant had to go inside to another square with similar dimensions (i.e., 3 × 3 m) situated 1 m (parallelly) to the first one. In this square, there were four numbers (1, 2, 3, and 4) placed on the ground (each number was situated on each corner of the square). The participant had to step on each number in ascending order (i.e., 1, 2, 3, and 4). The position of the numbers 2 and 3 was changed by the evaluator each time, so the participant could not know how the numbers were situated until they arrived at the square. Notice that the numbers 4, and 1 were always kept in the same place. The reason for keeping numbers 4 and 1 in the same place, was to be sure that all the participants started and finished in the same place in this section, so they had to complete the same distance until they reached the next task. Once the participant stepped on the number 4, he had to walk to the next station, which was situated 1 m (in parallel) from the last number.

#### Locomotor section

Subsequently, the participant started the second section of the test. Notice that participants did not interrupt their movement at the onset of this section; instead, the researcher registered the initiation time as participants crossed the designated starting line. A similar procedure was followed when the third section started. In this section, they had to jump 4 fences (height 20 cm), situated at a distance of 2 m between them. Afterward, they had to complete a zigzag task composed of five cones (6 m long × 3 m wide). Then, another zigzag had to be completed, but at this time each cone had a stick inside (1 m height) and the task consisted of going through the cones (separated by a 1 m among them) without touching any cone. When the last cone was passed, the older adult had to move to the third section, which was placed 1 m after the last cone.

#### Manipulative section

In the last section, the older adult had to pick up a ball from the ground and throw it into two hoops placed to the right and left side of the participant (0.5 m apart). After, the participant had to put the ball on the ground and carry the ball by dribbling with the feet until crossing the line that indicated the end of the test. Notice that to complete the test, the ball, and the participant had to cross the finish line since the chronometer was stopped when both (ball and the participant) crossed the finish line.

#### Procedure

Before starting the test, the evaluator carefully explained each section of the test, performing the section himself. After that, a familiarization trial was allowed. Once the participants understood properly each section of the test, the assessment started. Notice that a self-selected pace was allowed; however, the evaluator explained that the test should be completed as fast as possible. Each participant completed the test individually in a separate room to minimize observer bias, while the remaining participants waited in a designated area until their turn. A verbal encouragement was given by the evaluator to keep the motivation and complete the test properly. Two attempts were allowed, and the best one was registered for statistical analysis. If some Sects. (1, 2, or 3) were not completed adequately, the test was considered null, and a new attempt was allowed. The assessment was conducted on a flat surface in a quiet environment, with the temperature maintained between 24 and 26 °C throughout the procedure. The test was repeated 1 week later, in the same place maintaining the same environmental condition, by 100 older adults to assess the reliability of the test (test–retest).

### Statistical analysis

Data were analyzed using SPSS (version 25.0) for Windows (Spss Inc., Chicago, USA), establishing the level of significance in *p* < 0.05. Before conducting the analysis, the normal distribution and homogeneity tests (Kolmogorov–Smirnov and Levene’s test, respectively) were performed. The findings are displayed as means, and standard deviations (SD). Validity and reliability of the MC test were evaluated with relative reliability (test–retest consistency) and absolute reliability using both, the standard error of measurement (SEM) and the minimum detectable change (MDC). The SEM was calculated as the SD of the mean differences between the test and retest divided by √2 [40]. The quality of the SEM was categorized as “very good” < 5% of the total score, “good”, between 5 and 10%, “doubtful”, between 10 and 20%, and “negative”, above 20% [41]. The MDC establishes the limits within which changes in the measurement score can be attributed to measurement error. It is calculated as 1.96 * √2 * SEM [42]. To express SEM and MDC as percentages, the following formula was used: SEM% or MDC% = (SEM or MDC/mean) × 100, where the mean is the average of the test and retest scores [43]. In addition, relative reliability was evaluated using the intraclass correlation coefficient (ICC) and Pearson’s correlation. Bland–Altman plot was used to show agreement between the test and retest (1 week later), also to detect outliers. A 95% confidence interval (CI) of the mean difference was used to identify systematic biases. In addition, a partial correlation analysis adjusted for age and sex was carried out between the different sections of the test. Moreover, content validity was conducted by five experts in physical activity and sports sciences, and using the Delphi method evaluated the test structure on a Likert scale from 1 to 5, ranging from not relevant to highly relevant, assessing the characteristics of congruence, relevance, and pertinence. The degree of agreement among experts was determined using Fleiss’ kappa coefficient.

## Results

Regarding the content validity of the test, a Fleiss’ Kappa coefficient of 0.811 was obtained**.** In relation to absolute reliability, SEM values were 0.762 (1.01%), 0.791 (2.69%), 0.949 (5.16%), and 1.524 (1.79%) for Sections 1, 2, 3, and total test time, respectively. Also, MDC values were 2.112 (5.64%), 2.192 (7.47%), 2.630 (14.31%), and 4.224 (4.96%) for Sections 1, 2, and 3, respectively. Regarding ICC values, the test showed 0.959 (95% confidence interval [CI] = 0.948–0.969; *p* < 0.001) in Section 1; 0.995 (95% CI = 0.992–0.996, *p* < 0.001) in Section 2; 0.989 (95% CI = 0.983–0.992; *p* < 0.001) in Section 3; and 0.997 (95% CI = 0.996–0.998; *p* < 0.001) for the total test. Additionally, partial correlation analysis showed a strong correlation between the time of the total test and with time needed to complete Section 1 (*r* = 0.919, *p* < 0.001), Section 2 (*r* = 0.952, *p* < 0.001), and Section 3 (*r* = 0.851, *p* < 0.001). Moreover, a strong correlation between Section 1 and Section 2 (*r* = 0.844, *p* < 0.001), also Sections 1 and 3 (*r* = 0.647, *p* < 0.001), and between Sections 2 and Section 3 (*r* = 0.743, *p* < 0.001) was found. A weak correlation was found between age with total time (*r* = 0.214, *p* = 0.001), Sect. 1 (*r* = 0.244, *p* < 0.001), and Section 2 (*r* = 0.237, *p* < 0.001).

**Fig. 2:**
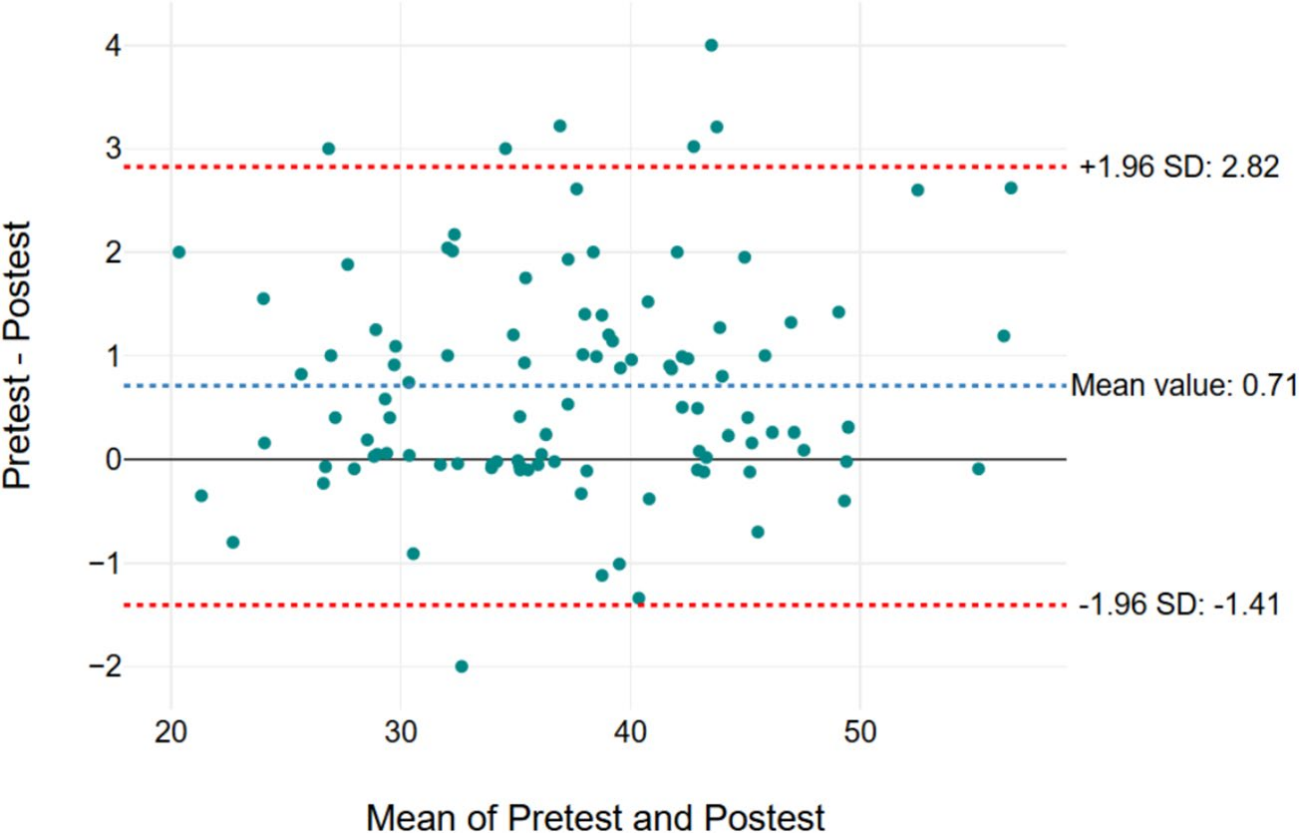
Bland–Altman graph results of Section 1. The x-axis means pre-test and post-test values (Sections 1, 2, 3, and total test) and the y-axis indicates the difference values for the test-re-test (Sections 1, 2, 3, and total test)

**Fig. 3:**
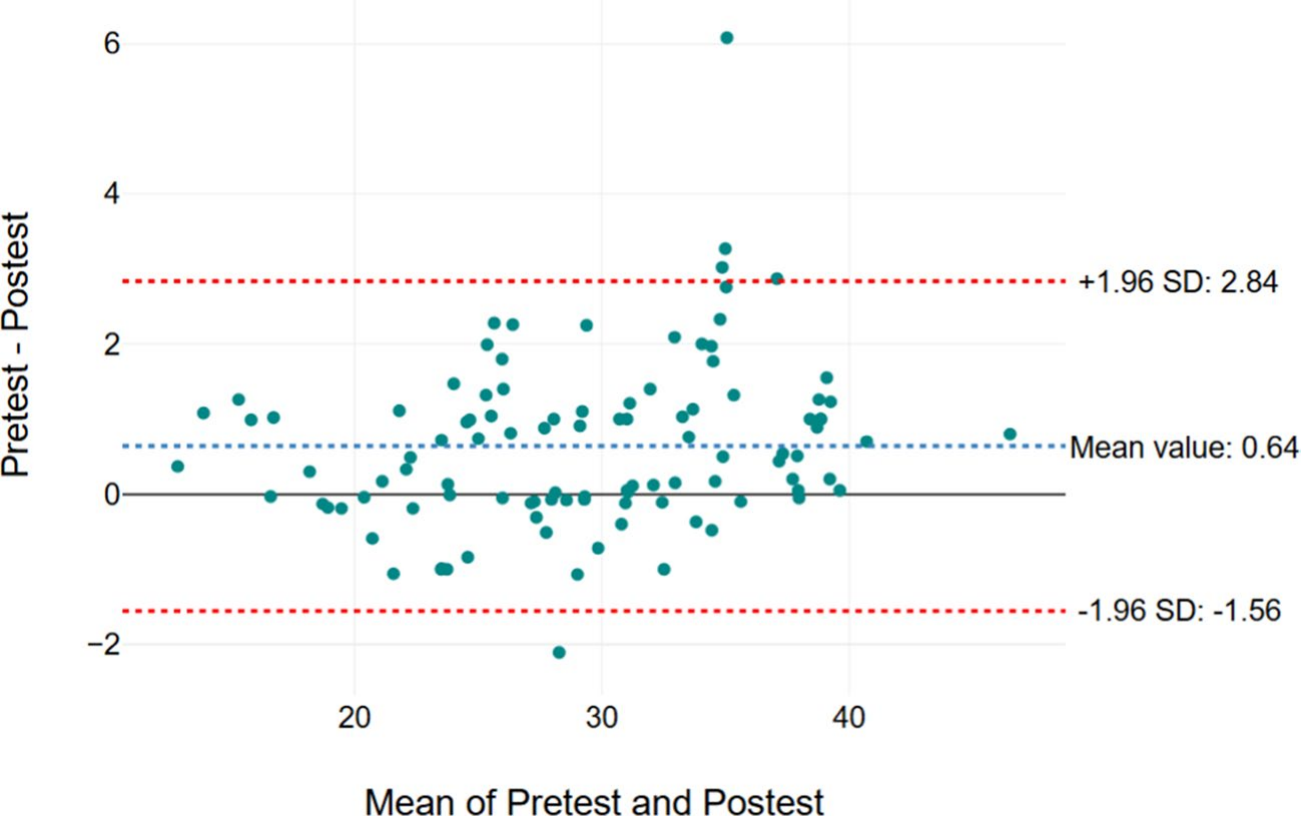
Bland–Altman graph results of Section 2. The x-axis means pre-test and post-test values (Sections 1, 2, 3, and total test) and the y-axis indicates the difference values for the test-re-test (Sections 1, 2, 3, and total test)

**Fig. 4:**
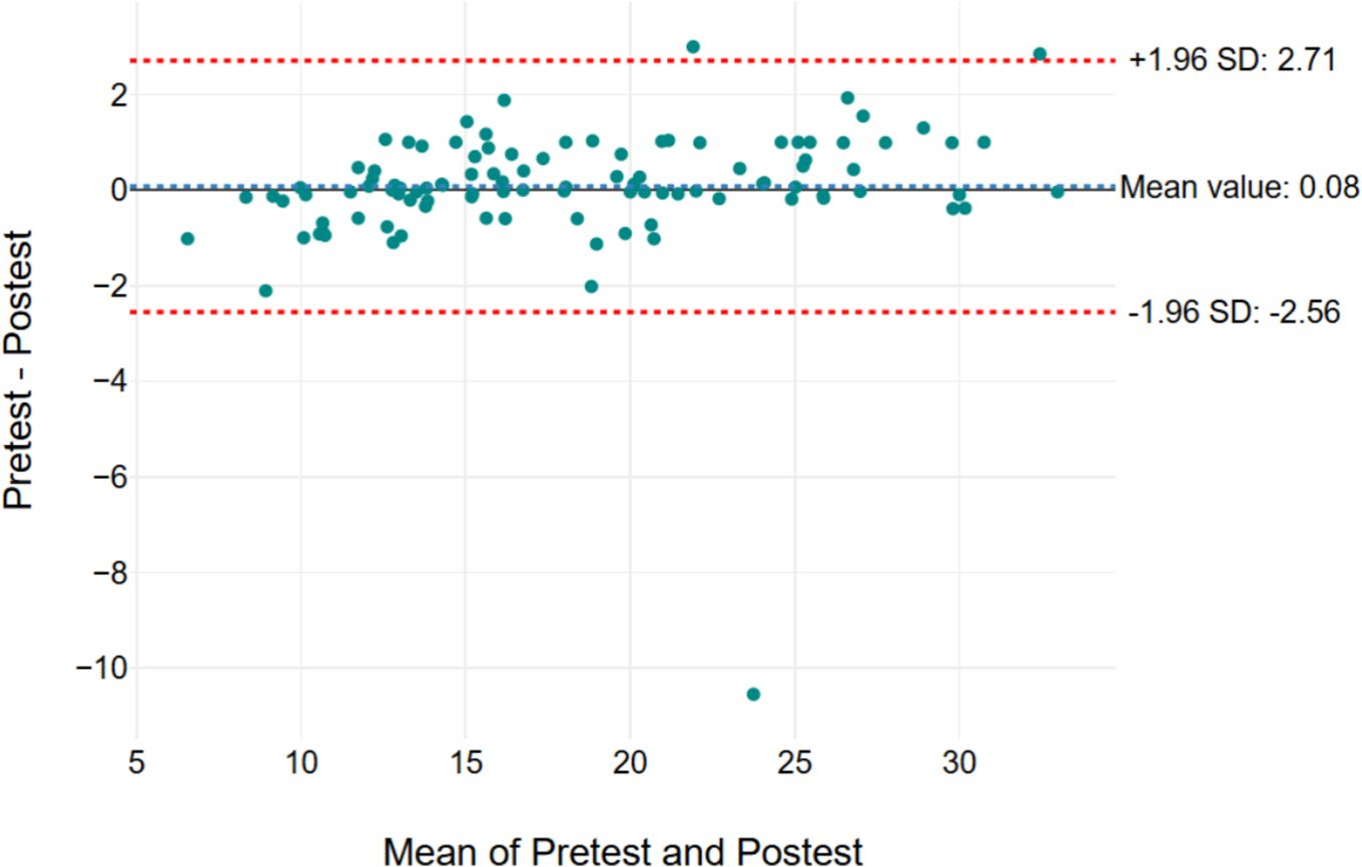
Bland–Altman graph results of section. The x-axis means pre-test and post-test values (Sections 1, 2, 3, and total test) and the y-axis indicates the difference values for the test-re-test (Sections 1, 2, 3, and total test)

**Fig. 5:**
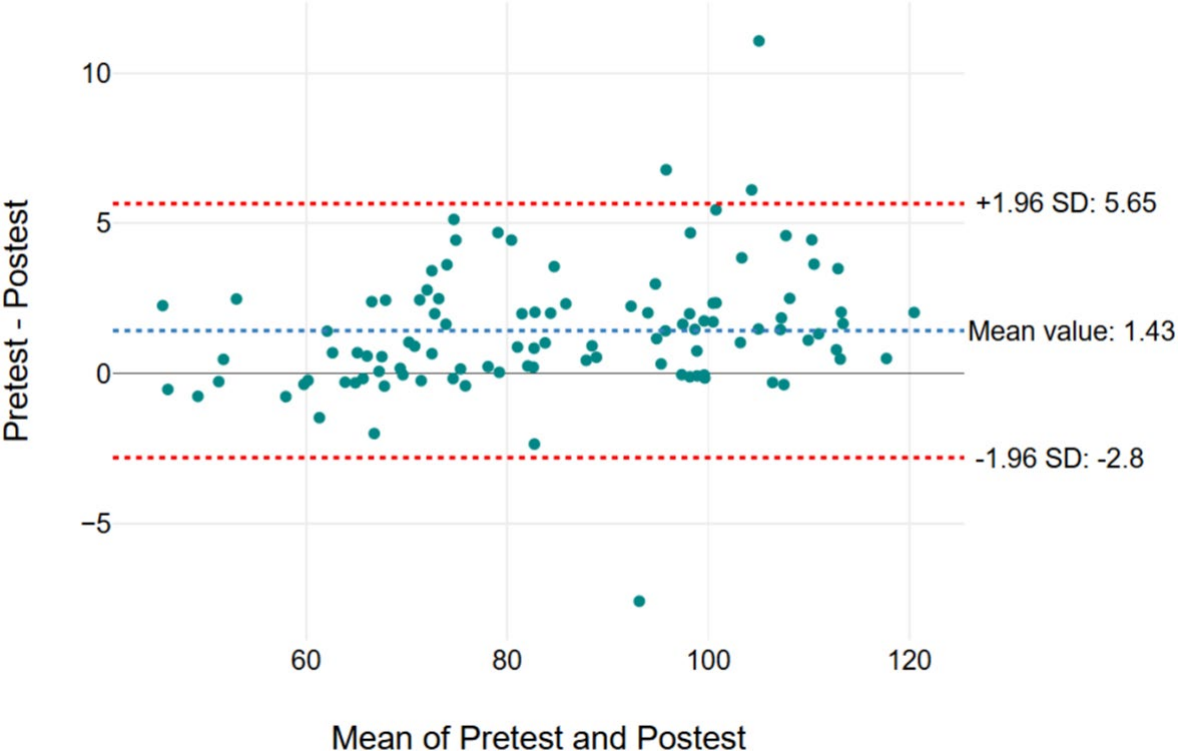
Bland–Altman graph results of total test. The x-axis means pre-test and post-test values (Sections 1, 2, 3, and total test) and the y-axis indicates the difference values for the test-re-test (Sections 1, 2, 3, and total test)

Figures 2, 3, 4, and 5 show the Bland–Altman graph, respectively. It showed limits of agreement (2 SD) of 2.82 s/− 1.41 s in Secti MDC/mean) × 100, where the mean is the 1 (Fig. 2), 2.84 s and − 1.56 s in Section 2 (Fig. 3), 2.71 s and − 0.2.56 s in Section 3 (Fig. 4), and 5.65 s and − 2.8 s in total test (Fig. 5). The result of this test indicated a good agreement between the test and retest sessions.

Age, anthropometric data and the time to complete the HMCT-OA of the participants separated by sex and by age groups are listed in Table 1. No significant differences (*p* > 0.05) were found between sexes in the HMCT-OA performance; however, an effect of age was observed. Specifically, the group of individuals over 80 years old showed poorer performance in the Motor-Cognitive section (*p* < 0.001), the Locomotor section (*p* < 0.001), and the total test time (*p* < 0.001), compared to the other age groups.

**Table 1:** Anthropometric data of the participants separated by sex and age groups

	Total Mean (SD) (*n* = 238)	Men Mean (SD) (*n* = 66)	Women Mean (SD) (*n* = 172)	*p*-value	Group 1 (60–69 years) (*n* = 82)	Group 2 (70–79 years) (*n* = 125)	Group 3 (> 80 years) (*n* = 32)	*p*-value	Effect size (*η*_*p*_^*2*^*)*
Age (years)	72.43 (5.95)	71.65 (6.16)	72.33 (5.86)	0.211	66.37 (2.33)a	73.68 (2.80)b	82.96 (2.78)c	< 0.001	0.801
Weight (kg)	70.13 (10.58)	71.14 (8.94)	69.75 (11.14)	0.366	68.85 (11.96)	70.35 (9.74)	72.51 (9.58)	0.238	
Height (cm)	159.80 (6.47)	161.52 (6.49)	157.33 (6.46)	0.460	161.10 (6.70)	160.10 (6.70)	157.90 (8.07)	0.812	
BMI (kg/m^2^)	21.70 (3.71)	21.48 (3.57)	21.79 (3.77)	0.567	21.07 (4.27)a	21.79 (3.46)a,b	22.94 (2.67)b	0.048	0.025
Motor cognitive section (s)	41.74 (12.60)	39.52 (13.18)	42.59 (12.31)	0.077	40.75 (13.74)a	40.07 (10.50)a	50.75 (13.63)b	< 0.001	0.080
Locomotor section (s)	33.86 (12.21)	32.06 (12.37)	34.55 (12.11)	0.164	33.23 (12.21)a	31.51 (9.70)a	44.34 (13.58)b	< 0.001	0.117
Manipulative section (s)	21.19 (8.99)	19.73 (10.31)	21.75 (8.40)	0.144	21.33 (10.08)	20.31 (8.14)	24.25 (8.79)	0.098	
Total test (s)	96.80 (31.24)	91.33 (32.60)	98.90 (30.54)	0.082	95.41 (32.60)a	91.90 (25.47)a	119.35 (33.23)b	< 0.001	0.082

*WC* waist circumference, *BMI* body mass index, *Wom-Rest* Wom-Rest Recognition Test; G1, G2, and G3, age group 1, 2, and 3, respectively. In post hoc analysis, different subscript letters (a, b, c) indicate significant differences (*p* < 0.05) between age groups

## Discussion

The objective of this study was to design and analyze the test–retest reliability of a test to assess MC in older adults, as well as the capacity of this test to discriminate between sex and age. The results indicated adequate reliability for the HMCT-OA in older adults. Thus, this test is safe, easy to perform, and highly acceptable for this population. The HMCT-OA provides a simple and inexpensive tool to measure MC in older adults, and it might be valuable to perform comparative studies. Therefore, this test is already in line with the ecological approach regarding the use of motor tasks that are close to real-life conditions.

Nowadays, several tests allow to assess mobility, and balance in older adults (e.g., Timed Up and Go test, SPBB…) [32]. However, there is no universal consensus about which test can be considered as a “gold standard” to assess MC in older adults [44, 45]. In addition, most of these tests assess MC isolated, as a consequence, relying on a single type of assessment does not offer a thorough evaluation of overall MC levels and may limit the ability to effectively explain their relationship with various outcomes [29, 46]. On the contrary, the test presented in the current study allows assessing MC in older adults with a holistic perspective since it has been designed emphasizing on perceptuomotor integration under dynamic performance conditions (e.g., decision-making, force regulation, speed, and accuracy…) [29]. Furthermore, the current test was designed following the requirements to be considered with ecological validity (i.e., include decision-making action (Section 1), speed, and accuracy (Section 2)…) [47].

A second objective was to examine the influence of age and sex on the HMCT-OA performance. In the current study, no effect of sex was observed; nevertheless, age did have an effect on HMCT-OA performance. The effect of age on the decline of MC is intrinsic to the aging process. Previous studies [27, 48, 49] showed an age-related deterioration in physical functioning, physical fitness, balance, gait speed, obstacle negotiation and performance in complex walking tasks. However, a key finding of the current study was that a significant decline in HMCT-OA performance becomes evident from the age of 80. Specifically, male and female participants aged 80 years and older required more time to complete the HMCT-OA, especially in the Motor-Cognitive section and the Locomotion section. Other previous studies highlight this chronological age as a critical point in the accelerated deterioration of motor performance associated with aging [27, 48, 50]. Similarly, Párraga and colleagues [27] found no effect of sex on linear and complex gait performance in variables such as speed and gait variability, but they did find a significant negative effect of age on these variables from the age of 80.

Poor performance on the HMCT-OA with age in older adults may be due to visual, orthopedic and cognitive decline that might contribute to the worsening of obstacle-crossing performance [51]. Moreover, with aging, gait progressively becomes less automatic and requires higher demands on already declining frontal-dependent executive control resources, which normally enable individuals to efficiently tackle mobility tasks [52]. In this regard, older adults display involvement of more widespread brain regions for motor control than young adults, principally the prefrontal cortex and basal ganglia networks [4]. Additionally, the reduction in muscle strength, along with changes in body fat percentage, flexibility, agility, and endurance [53] associated with increased sedentary behavior may help explain the onset of functional limitations and, consequently, the decline in MC, which becomes particularly relevant from the age of 80 onwards due to increased multimorbidity [48, 54].

Finally, regarding the absence of sexual dimorphism in the HMCT-OA performance, the findings of the current study do not support the previous research. In this regard, Latorre et al. [55] noted that men showed greater performance than women in a complex gait test, although no significant differences between sexes for any age group were found. Likewise, Jiménez-Jiménez et al. [56] and Rudisill et al. [57] showed that men show higher performance in various MC tasks. Sex may influence adaptive walking across locomotor tasks such as obstacle crossing and could manifest in a greater risk of mobility impairment in women due to a conservative adaptive walking pattern [58]. Moreover, sex differences in MC could be related to daily physical activity levels, as previous research has shown a decline in women's physical activity levels with age compared to men [59, 60]. Also, women worsened than men in relation to quality of life, pain, depression, number of falls and executive function [59, 61, 62]. It could cause for the differences in physical functioning between women and men where older women have more limitations than older men [63] and the women’s self-perception regarding mobility in subjective measure [64]. However, the present findings seem to be consistent with previous research [65, 66] which found no differences between both sex in motor performance: gait speed, stability. The contradictory results of different studies could be related to the sociodemographic and cultural characteristics of the participants, such as their level of physical activity. Therefore, more research on this topic needs to be undertaken.

Some limitations of this study must be mentioned. The main limitation is the cross-sectional design; MC performance in aging should be measured in a longitudinal study. Second, the sample included older adults in southern Spain, and generalization to a wider population should be made with caution. Finally, other factors, such as the participant’s level of physical activity, physical fitness, and cognitive status, were not recorded and could help explain some of the findings in this study. Despite these limitations, the strength of this study is that it provides a new test to assess MC in the older population, which could be used in other studies that should increase the number of participants analyzed and provide reference values by sex and age.

### Practical applications

The aging process is characterized by the presence of high interindividual variation between individuals of the same chronological age prompting a search for biomarkers that capture this heterogeneity [67]. People of the same age may not age at the same rate. Quantitative biomarkers of aging are valuable tools to measure physiological age, evaluate the extent of “healthy aging”, and potentially predict health span and life span for an individual: Physical function and anthropometry are the most practical measurements among phenotypic biomarkers of aging [9]. Especially, measuring physical capacity is a feasible method to identify accelerated aging and biological age [10]. Functional assessments for physical performance such as handgrip strength, chair stand, gait speed, complex gait, timed up and go, standing balance times, and six-minute walk tests are frequently used for monitoring the biological aging process and are predictors of all-cause mortality and survival in older community-dwelling populations [11, 12, 55, 68]. With this in mind and from a practical point of view, MC analyzed ecologically and holistically through the HMCT-OA, could be another valid phenotypic biomarker for assessing the aging process.

## Conclusion

In conclusion, the results of the current study showed very good reliability test–retest parameters for the HMCT-OA in older adults. The test is safe, easy to administer, acceptable, and appropriate for older adults, and it effectively discriminates between age groups within a healthy older adults’ population. Consequently, teachers, coaches, physicians, and other professionals working with individuals in this age group may employ this test to assess motor competence, even in settings with limited materials and technological resources.

## Data Availability

The data that support the findings of this study are available from the corresponding author upon reasonable request.

## References

[1] “Demography: interactive publication 2024 edition - News articles - Eurostat.” Accessed: Feb. 10, 2025. [Online]. Available: https://ec.europa.eu/eurostat/web/products-eurostat-news/w/wdn-20240515-1.

[2] Beard JR, et al. The World report on ageing and health: a policy framework for healthy ageing. Lancet. 2016;387(10033):2145–54. 10.1016/S0140-6736(15)00516-4. 26520231 10.1016/S0140-6736(15)00516-4 PMC4848186

[3] Rittweger J, Kwiet A, Felsenberg D. Physical performance in aging elite athletes–challenging the limits of physiology. J Musculoskelet Neuronal Interact. 2004;4(2):159–60. 15615117

[4] Seidler RD et al. “Motor control and aging: links to age-related brain structural, functional, and biochemical effects,” 2010. 10.1016/j.neubiorev.2009.10.005. 10.1016/j.neubiorev.2009.10.005 PMC2838968 19850077

[5] Hunter SK, Pereira XHM, Keenan KG. The aging neuromuscular system and motor performance. J Appl Physiol. 2016;121(4):982–95. 10.1152/JAPPLPHYSIOL.00475.2016. 27516536 10.1152/japplphysiol.00475.2016 PMC5142309

[6] Frolov NS, et al. Age-related slowing down in the motor initiation in elderly adults. PLoS ONE. 2020;15(9):e0233942. 10.1371/JOURNAL.PONE.0233942. 32937652 10.1371/journal.pone.0233942 PMC7494367

[7] Leversen JSR, Haga M, Sigmundsson H. From children to adults: motor performance across the life-span. PLoS One. 2012. 10.1371/journal.pone.0038830. 22719958 10.1371/journal.pone.0038830 PMC3377693

[8] Sigmundsson H, Lorås H, Haga M. Assessment of motor competence across the life span: aspects of reliability and validity of a new test battery. Sage Open. 2016. 10.1177/2158244016633273.

[9] Xia X, Chen W, McDermott J, Han J-DJ. Molecular and phenotypic biomarkers of aging. F1000Res. 2017. 10.12688/f1000research.10692.1. 28663789 10.12688/f1000research.10692.1 PMC5473407

[10] Tzemah-Shahar R, Hochner H, Iktilat K, Agmon M. What can we learn from physical capacity about biological age? A systematic review. Ageing Res Rev. 2022;77:101609. 35306185 10.1016/j.arr.2022.101609

[11] Studenski S. Gait speed and survival in older adults. JAMA. 2011;305(1):50–8. 10.1001/jama.2010.1923. 21205966 10.1001/jama.2010.1923 PMC3080184

[12] Wagner KH, Cameron-Smith D, Wessner B, Franzke B. Biomarkers of aging: from function to molecular biology. Nutrients. 2016;8(6): 338. 10.3390/NU8060338. 27271660 10.3390/nu8060338 PMC4924179

[13] Robinson LE, et al. Motor competence and its effect on positive developmental trajectories of health. Sports Med. 2015;45(9):1273–84. 10.1007/S40279-015-0351-6/FIGURES/2. 26201678 10.1007/s40279-015-0351-6

[14] Stodden DF, et al. A developmental perspective on the role of motor skill competence in physical activity: an emergent relationship. Quest. 2008;60(2):290–306. 10.1080/00336297.2008.10483582.

[15] Logan SW, KiplingWebster E, Getchell N, Pfeiffer KA, Robinson LE. Relationship between fundamental motor skill competence and physical activity during childhood and adolescence: a systematic review. Kinesiol Rev. 2015;4(4):416–26. 10.1123/KR.2013-0012.

[16] Cattuzzo MT et al. “Motor competence and health related physical fitness in youth: a systematic review,”. 2016. Elsevier Ltd. 10.1016/j.jsams.2014.12.004. 10.1016/j.jsams.2014.12.004 25554655

[17] Barros WMA, et al. Effects of overweight/obesity on motor performance in children: a systematic review. Front Endocrinol (Lausanne). 2022;12:759165. 10.3389/FENDO.2021.759165/BIBTEX. 35126307 10.3389/fendo.2021.759165 PMC8812008

[18] Batez M, Milošević Ž, Mikulić I, Sporiš G, MačAk D, Trajković N. Relationship between motor competence, physical fitness, and academic achievement in young school-aged children. Biomed Res Int. 2021;2021(1):6631365. 10.1155/2021/6631365. 33628796 10.1155/2021/6631365 PMC7884140

[19] Barnes JN. Exercise, cognitive function, and aging. Adv Physiol Educ. 2015. 10.1152/advan.00101.2014. 26031719 10.1152/advan.00101.2014 PMC4587595

[20] Motl RW, McAuley E. “Physical activity, disability, and quality of life in older adults,” 2010. 10.1016/j.pmr.2009.12.006. 10.1016/j.pmr.2009.12.006 20494278

[21] Vogel T, Brechat PH, Lepr??tre PM, Kaltenbach G, Berthel M, Lonsdorfer J. “Health benefits of physical activity in older patients: a review,” 2009. 10.1111/j.1742-1241.2008.01957.x. 10.1111/j.1742-1241.2008.01957.x 19196369

[22] Erickson KI, Hillman CH, Kramer AF. “Physical activity, brain, and cognition,” 2015. 10.1016/j.cobeha.2015.01.005.

[23] Paterson DH, Warburton DE. “Physical activity and functional limitations in older adults: a systematic review related to Canada’s Physical Activity Guidelines,” 2010. [Online]. Available: http://www.ijbnpa.org/content/7/1/38. 10.1186/1479-5868-7-38 PMC2882898 20459782

[24] Gruszczyński N, Perkowski R. Assessment of balance and risk of falls in people over 60 years old. J Edu Health Sport. 2024;61: 51833. 10.12775/jehs.2024.61.51833.

[25] Lopes L, et al. A narrative review of motor competence in children and adolescents: what we know and what we need to find out. Int J Environ Res Public Health. 2021;15:18. 10.3390/ijerph1801. 10.3390/ijerph18010018 PMC7792958 33375134

[26] De Meester A et al. “The relationship between actual and perceived motor competence in children, adolescents and young adults: a systematic review and meta-analysis,”. 2020. Springer Science and Business Media Deutschland GmbH. 10.1007/s40279-020-01336-2. 10.1007/s40279-020-01336-2 32970291

[27] Párraga-Montilla J, Pozuelo-Carrascosa D, Carmona-Torres J, Laredo-Aguilera J, Cobo-Cuenca A, Latorre-Román P. Gait performance as an indicator of cognitive deficit in older people. Int J Environ Res Public Health. 2021. 10.3390/ijerph18073428. 33806244 10.3390/ijerph18073428 PMC8037000

[28] Tiernan C, Schwarz DJ, Goldberg A. Associations of usual and fast gait speed with physical performance and balance confidence in community-dwelling older adults: implications for assessment. J Geriatric Phys Ther. 2023. 10.1519/jpt.0000000000000397. 10.1519/JPT.0000000000000397 37820362

[29] Hulteen RM et al. “Reinvest to assess: advancing approaches to motor competence measurement across the lifespan,” Springer Science and Business Media Deutschland GmbH. 2023. 10.1007/s40279-022-01750-8. 10.1007/s40279-022-01750-8 35997861

[30] Martins C, et al. Motor competence and body mass index in the preschool years: a pooled cross-sectional analysis of 5545 children from eight countries. Sports Med. 2024;54(2):505–16. 10.1007/S40279-023-01929-7/FIGURES/2. 37747664 10.1007/s40279-023-01929-7 PMC10939976

[31] den Uil AT, Janssen M, Busch V, Kat I, Scholte RHJ. “The relationships between children’s motor competence, physical activity, perceived motor competence, physical fitness and weight status in relation to age,” PLoS One, 2023;18(4). 10.1371/journal.pone.0278438. 10.1371/journal.pone.0278438 PMC10104338 37058506

[32] Soubra R, Chkeir A, Novella JL. “A systematic review of thirty-one assessment tests to evaluate mobility in older adults,” 2019, Hindawi Limited. 10.1155/2019/1354362. 10.1155/2019/1354362 PMC6610744 31321227

[33] Tinetti ME. Performance-oriented assessment of mobility problems in elderly patients. J Am Geriatr Soc. 1986;34(2):119–26. 10.1111/J.1532-5415.1986.TB05480.X. 3944402 10.1111/j.1532-5415.1986.tb05480.x

[34] Podsiadlo D, Richardson S. The timed ‘up & go’: a test of basic functional mobility for frail elderly persons. J Am Geriatr Soc. 1991;39(2):142–8. 10.1111/J.1532-5415.1991.TB01616.X. 1991946 10.1111/j.1532-5415.1991.tb01616.x

[35] Andrade-Lara K, Cabrera Linares J, PárragaMontilla J, Latorre Román P. “Factors influencing gait performance in older adults in a dual-task paradigm,” Geroscience, 2024;1–13. 10.1007/s11357-023-01052-5. 10.1007/s11357-023-01052-5 PMC11009214 38190081

[36] Hulteen RM, et al. Reinvest to assess: advancing approaches to motor competence measurement across the lifespan. Sports Med. 2022;53(1):33–50. 10.1007/S40279-022-01750-8. 35997861 10.1007/s40279-022-01750-8

[37] Bardid F, Vannozzi G, Logan SW, Hardy LL, Barnett LM. “A hitchhiker’s guide to assessing young people’s motor competence: deciding what method to use,” 2019. Elsevier Ltd. 10.1016/j.jsams.2018.08.007. 10.1016/j.jsams.2018.08.007 30166086

[38] S. Xuekelati, Z. Maimaitiwusiman, … H. X.-E., and undefined 2024, “Handgrip strength: a simple and effective tool to recognize decreased intrinsic capacity in Chinese older adults,” ExpGerontol, 2024;196:112567. Accessed: 1 Feb 2025. [Online]. Available: https://www.sciencedirect.com/science/article/pii/S0531556524002134. 10.1016/j.exger.2024.112567 39236871

[39] Wang L-Y et al. “Physical activity as a predictor of activities of daily living in older adults: a longitudinal study in China,” frontiersin.orgLY Wang, HX Chen, H Zhu, ZY Hu, CF Zhou, XY HuFrontiers in Public Health, 2024•frontiersin.org, 2024. 10.3389/fpubh.2024.1444119. 10.3389/fpubh.2024.1444119 PMC11543459 39525460

[40] Weir JP. “Quantifying test-retest reliability using the intraclass correlation coefficient and the SEM,” 2005. [Online]. Available: http://journals.lww.com/nsca-jscr. 10.1519/15184.1 15705040

[41] Streiner D, Norman G. Health measurement scales: a practical guide to their development and their use. Aust N Z J Public Health. 2008;40(3):294–5. 10.1111/1753-6405.12484. 10.1111/1753-6405.12484 27242256

[42] Stratford P. Getting more from the literature: estimating the standard error of measurement from reliability studies. Physiother Can. 2004;56(01):027. 10.2310/6640.2004.15377.

[43] Leon-Llamas JL, Villafaina S, Murillo-Garcia A, Domínguez-Muñoz FJ, Gusi N. Test–retest reliability and concurrent validity of the 3 m backward walk test under single and dual-task conditions in women with fibromyalgia. J Clin Med. 2022;12(1):212. 36615014 10.3390/jcm12010212 PMC9821607

[44] Rubio Castañeda FJ, Tomás Aznar C, MuroBaquero C, Chico Guerra J. “Descripción de los instrumentos de medida de la movilidad en personas mayores de 65 años. Revisión Sistemática.,” 2015. 10.4321/S1135-57272015000600003 26786303

[45] Logan SW, Barnett LM, Goodway JD, Stodden DF. Comparison of performance on process- and product-oriented assessments of fundamental motor skills across childhood. J Sports Sci. 2017;35(7):634–41. 10.1080/02640414.2016.1183803. 27169780 10.1080/02640414.2016.1183803

[46] True L, Brian A, Goodway J, Stodden D. Relationships between product and process-oriented measures of motor competence and perceived competence. J Mot Learn Dev. 2017;5(2):319–35. 10.1123/jmld.2016-0042.

[47] Hulteen RM, Morgan PJ, Barnett LM, Stodden DF, Lubans DR. Development of foundational movement skills: a conceptual model for physical activity across the lifespan. Sports Med. 2018;48(7):1533–40. 10.1007/s40279-018-0892-6. 29524160 10.1007/s40279-018-0892-6

[48] Santos M, Campos E, Párraga-Montilla J, Aragón-Vela J, Latorre-Román P. Effects of a functional training program in patients with fibromyalgia: a 9-year prospective longitudinal cohort study. Scand J Med Sci Sports. 2020;30(5):904–13. 10.1111/SMS.13640. 32077144 10.1111/sms.13640

[49] De Cock AM, et al. Gait characteristics under different walking conditions: association with the presence of cognitive impairment in community-dwelling older people. PLoS One. 2017;12(6): e0178566. 10.1371/JOURNAL.PONE.0178566. 28570662 10.1371/journal.pone.0178566 PMC5453541

[50] Muir BC, Rietdyk S, Haddad JM. Gait initiation: the first four steps in adults aged 20–25 years, 65–79 years, and 80–91 years. Gait Posture. 2014;39(1):490–4. 10.1016/j.gaitpost.2013.08.037. 24074729 10.1016/j.gaitpost.2013.08.037

[51] Galna B, Peters A, Murphy AT, Morris ME. Obstacle crossing deficits in older adults: a systematic review. Gait Posture. 2009;30(3):270–5. 10.1016/J.GAITPOST.2009.05.022. 19625191 10.1016/j.gaitpost.2009.05.022

[52] Forte R, et al. How older adults cope with cognitive complexity and environmental constraints during dual-task walking: the role of executive function involvement. Int J Environ Res Public Health. 2019;16(10): 1835. 10.3390/IJERPH16101835. 31126116 10.3390/ijerph16101835 PMC6571728

[53] Milanović Z, Pantelić S, Trajković N, Sporiš G, Kostić R, James N. Age-related decrease in physical activity and functional fitness among elderly men and women. Clin Interv Aging. 2013;8:549. 10.2147/CIA.S44112. 23723694 10.2147/CIA.S44112 PMC3665513

[54] Jindai K, Nielson CM, Vorderstrasse BA, Quiñones AR. Multimorbidity and functional limitations among adults 65 or older, NHANES 2005–2012. Prev Chronic Dis. 2016;13(11):E151. 10.5888/PCD13.160174. 27809419 10.5888/pcd13.160174 PMC5094859

[55] Latorre-Román PÁ, Carmona-Torres JM, Cobo-Cuenca AI, Laredo-Aguilera JA. Physical activity, ability to walk, weight status, and multimorbidity levels in older Spanish people: the national health survey (2009–2017). Int J Environ Res Public Health. 2020;17(12): 4333. 32560442 10.3390/ijerph17124333 PMC7344667

[56] Jiménez-Jiménez FJ, et al. Influence of age and gender in motor performance in healthy subjects. J Neurol Sci. 2011;302(1–2):72–80. 10.1016/J.JNS.2010.11.021. 21183189 10.1016/j.jns.2010.11.021

[57] Rudisill ME, Toole T. “Gender differences in motor performance of 50- to 79-year-old adults,” 1993;77(3 Pt 1): 939–947. 10.2466/PMS.1993.77.3.939. 10.2466/pms.1993.77.3.939 8284181

[58] Raffegeau TE, Kellaher GK, Terza MJ, Roper JA, Altmann LJ, Hass CJ. Older women take shorter steps during backwards walking and obstacle crossing. Exp Gerontol. 2019;122:60–6. 10.1016/j.exger.2019.04.011. 31034865 10.1016/j.exger.2019.04.011 PMC6771276

[59] Lepsy E, et al. Association of physical fitness with quality of life in community-dwelling older adults aged 80 and over in Poland: a cross-sectional study. BMC Geriatr. 2021. 10.1186/s12877-021-02421-5. 34503463 10.1186/s12877-021-02421-5 PMC8427892

[60] Mosallanezhad Z, Hörder H, Salavati M, Nilsson-Wikmar L, Frändin K. Physical activity and physical functioning in Swedish and Iranian 75-year-olds - a comparison. Arch Gerontol Geriatr. 2012;55(2):422–30. 10.1016/j.archger.2012.02.007. 22425242 10.1016/j.archger.2012.02.007

[61] Crimmins EM, Kim JK, Solé-Auró A. Gender differences in health: results from SHARE, ELSA and HRS. Eur J Public Health. 2011;21:81–91. 10.1093/eurpub/ckq022. 20237171 10.1093/eurpub/ckq022 PMC3023013

[62] Börsch-Supan A, Brugiavini A, Jürges H, Mackenbach J, Siegrist J, Weber G. “Health, ageing and retirement in Europe first results from the survey of health, ageing and retirement in Europe,” https://iris.unive.it/handle/10278/5086727.

[63] Stalling I, Gruber M, Bammann K. Sex differences in physical functioning among older adults: cross-sectional results from the OUTDOOR ACTIVE study. BMC Public Health. 2024;24(1):1766. 10.1186/s12889-024-19218-x. 38956507 10.1186/s12889-024-19218-x PMC11221023

[64] Halaweh H, Willen C, Grimby-Ekman A, Svantesson U. Physical activity and health-related quality of life among community dwelling elderly. J Clin Med Res. 2015;7(11):845–52. 10.14740/jocmr2307w. 26491496 10.14740/jocmr2307w PMC4596265

[65] Musselman K, Brouwer B. Gender-related differences in physical performance among seniors. J Aging Phys Act. 2005;13(3):239–53. 10.1123/JAPA.13.3.239. 16192652 10.1123/japa.13.3.239

[66] Dommershuijsen LJ, et al. Gait speed reference values in community-dwelling older adults – cross-sectional analysis from the Rotterdam study. Exp Gerontol. 2022;158: 111646. 10.1016/J.EXGER.2021.111646. 34861357 10.1016/j.exger.2021.111646

[67] McCrory C, et al. GrimAge outperforms other epigenetic clocks in the prediction of age-related clinical phenotypes and all-cause mortality. J Gerontol: Series A. 2021;76(5):741–9. 10.1093/gerona/glaa286 PMC8087266 33211845

[68] Cooper R, Kuh D, Hardy R. Objectively measured physical capability levels and mortality: systematic review and meta-analysis. BMJ (Online). 2010;341(7774):639. 10.1136/bmj.c4467. 10.1136/bmj.c4467 PMC2938886 20829298

